# Cure for Drunkenness

**Published:** 1858-07

**Authors:** 


### CURE FOR DRUNKENNESS.

No one who has once been a drunkard is ever safe from falling, this side the grave; it is a terrible truth, but it is a reality. He only is comparatively safe, who is in constant fear of falling.

We knew a Mrs. H., in our childhood, who finding her husband dead drunk one day, sewed him up in a sheet, and gave him a tremendous cowhiding. He never got drunk again; in this case it was the fear of the hide, and not of the fall. But one of the speediest rousers from a state of stupid, beastly intoxication we have ever read of is, to turn the brute on his right side, hold up his left arm, and pour a pitcher of cold water down his sleeve slowly. He will walk perfectly sober in five minutes. But this only cures until next time. We rather think that the raw hide is a more vivid remembrancer; and our old friend, cold water, must yield the palm this time.

---

A LABORIOUS and self-denying clergyman from Virginia, writes: "To your *February* article, 'POWER IN THE PULPIT,' I subscribe, as my venerable friend and countryman 'GRANT THORBURN' did to another on *The Bible and Materia Medica*, with my soul, body, and spirit. You have taken a right view of the subject, and I hope it will do great good."

